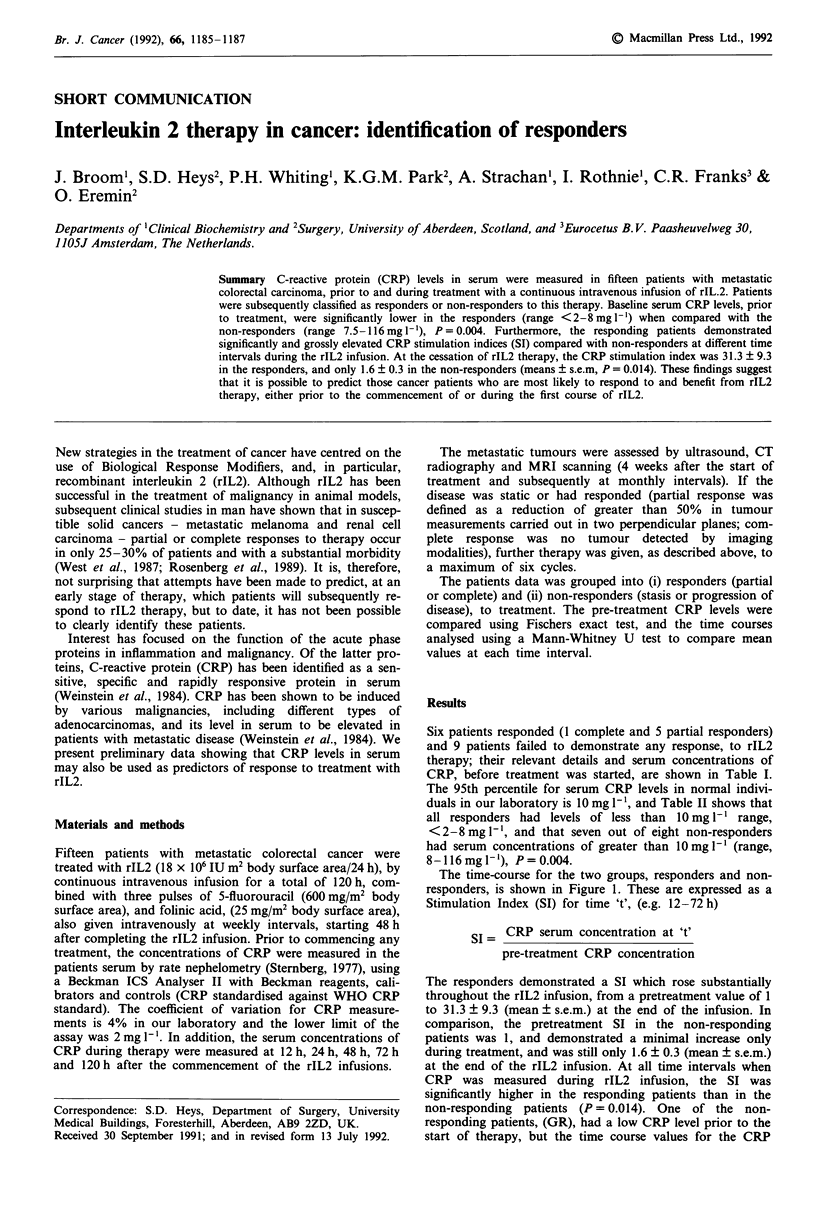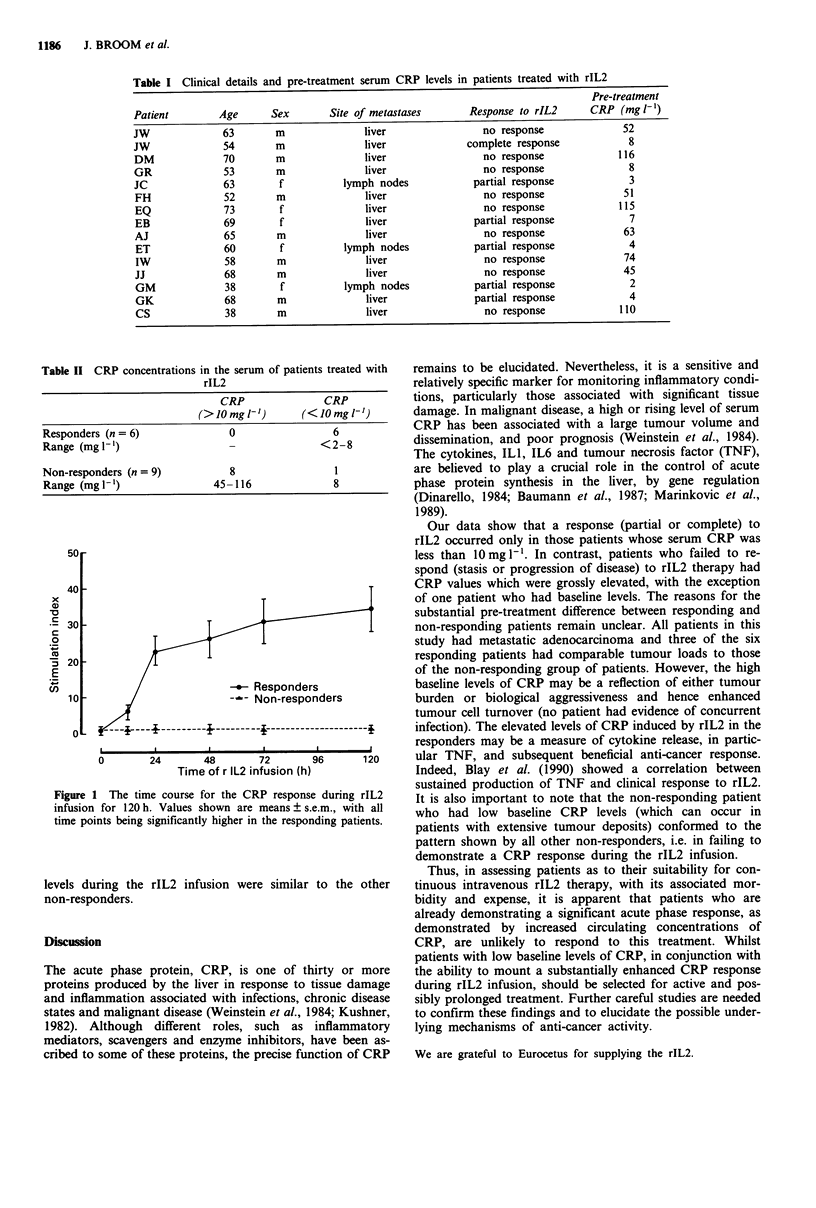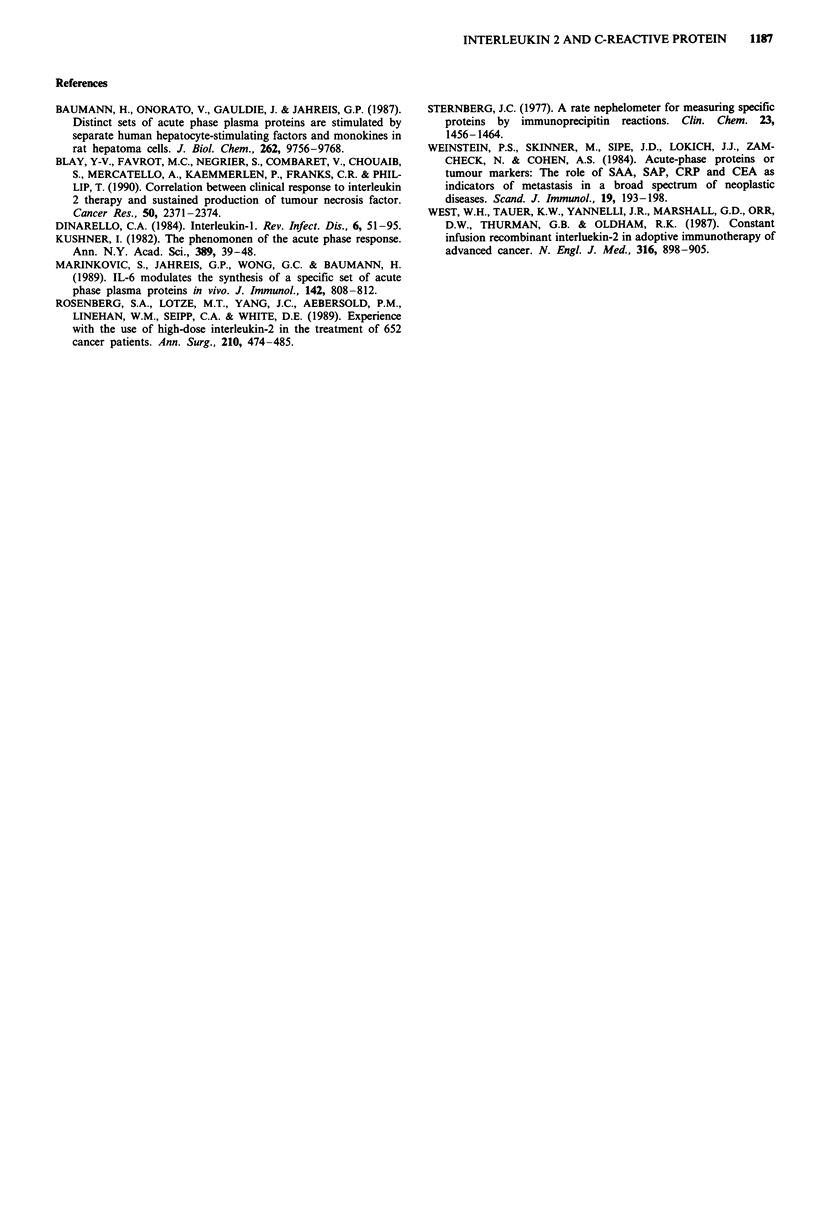# Interleukin 2 therapy in cancer: identification of responders.

**DOI:** 10.1038/bjc.1992.433

**Published:** 1992-12

**Authors:** J. Broom, S. D. Heys, P. H. Whiting, K. G. Park, A. Strachan, I. Rothnie, C. R. Franks, O. Eremin

**Affiliations:** Department of Clinical Biochemistry, University of Aberdeen, Scotland, UK.

## Abstract

C-reactive protein (CRP) levels in serum were measured in fifteen patients with metastatic colorectal carcinoma, prior to and during treatment with a continuous intravenous infusion of rIL.2. Patients were subsequently classified as responders or non-responders to this therapy. Baseline serum CRP levels, prior to treatment, were significantly lower in the responders (range < 2-8 mg l-1) when compared with the non-responders (range 7.5-116 mg l-1), P = 0.004. Furthermore, the responding patients demonstrated significantly and grossly elevated CRP stimulation indices (SI) compared with non-responders at different time intervals during the rIL2 infusion. At the cessation of rIL2 therapy, the CRP stimulation index was 31.3 +/- 9.3 in the responders, and only 1.6 +/- 0.3 in the non-responders (means +/- s.e.m, P = 0.014). These findings suggest that it is possible to predict those cancer patients who are most likely to respond to and benefit from rIL2 therapy, either prior to the commencement of or during the first course of rIL2.


Br. J. Cancer (1992), 66, 1185–1187 © Macmillan Press Ltd., 1992

SHORT COMMUNICATION

### Interleukin 2 therapy in cancer: identification of responders

J. Broom[1], S.D. Heys[2], P.H. Whiting[1], K.G.M. Park[2], A. Strachan[1], I. Rothnie[1], C.R. Franks[3] & O. Eremin[2]

*Departments of [1]Clinical Biochemistry and [2]Surgery, University of Aberdeen, Scotland, and [3]Eurocetus B.V. Paasheuvelweg 30, 1105J Amsterdam, The Netherlands.*

**Summary** C-reactive protein (CRP) levels in serum were measured in fifteen patients with metastatic colorectal carcinoma, prior to and during treatment with a continuous intravenous infusion of rIL.2. Patients were subsequently classified as responders or non-responders to this therapy. Baseline serum CRP levels, prior to treatment, were significantly lower in the responders (range $<2-8\ mg\,l^{-1}$) when compared with the non-responders (range $7.5-116\ mg\,l^{-1}$), $P = 0.004$. Furthermore, the responding patients demonstrated significantly and grossly elevated CRP stimulation indices (SI) compared with non-responders at different time intervals during the rIL2 infusion. At the cessation of rIL2 therapy, the CRP stimulation index was $31.3 \pm 9.3$ in the responders, and only $1.6 \pm 0.3$ in the non-responders (means ± s.e.m, $P = 0.014$). These findings suggest that it is possible to predict those cancer patients who are most likely to respond to and benefit from rIL2 therapy, either prior to the commencement of or during the first course of rIL2.

New strategies in the treatment of cancer have centred on the use of Biological Response Modifiers, and, in particular, recombinant interleukin 2 (rIL2). Although rIL2 has been successful in the treatment of malignancy in animal models, subsequent clinical studies in man have shown that in susceptible solid cancers – metastatic melanoma and renal cell carcinoma – partial or complete responses to therapy occur in only 25–30% of patients and with a substantial morbidity (West *et al.*, 1987; Rosenberg *et al.*, 1989). It is, therefore, not surprising that attempts have been made to predict, at an early stage of therapy, which patients will subsequently respond to rIL2 therapy, but to date, it has not been possible to clearly identify these patients.

Interest has focused on the function of the acute phase proteins in inflammation and malignancy. Of the latter proteins, C-reactive protein (CRP) has been identified as a sensitive, specific and rapidly responsive protein in serum (Weinstein *et al.*, 1984). CRP has been shown to be induced by various malignancies, including different types of adenocarcinomas, and its level in serum to be elevated in patients with metastatic disease (Weinstein *et al.*, 1984). We present preliminary data showing that CRP levels in serum may also be used as predictors of response to treatment with rIL2.

### Materials and methods

Fifteen patients with metastatic colorectal cancer were treated with rIL2 ($18 \times 10^6$ IU $m^2$ body surface area/24 h), by continuous intravenous infusion for a total of 120 h, combined with three pulses of 5-fluorouracil (600 mg/$m^2$ body surface area), and folinic acid, (25 mg/$m^2$ body surface area), also given intravenously at weekly intervals, starting 48 h after completing the rIL2 infusion. Prior to commencing any treatment, the concentrations of CRP were measured in the patients serum by rate nephelometry (Sternberg, 1977), using a Beckman ICS Analyser II with Beckman reagents, calibrators and controls (CRP standardised against WHO CRP standard). The coefficient of variation for CRP measurements is 4% in our laboratory and the lower limit of the assay was $2\ mg\,l^{-1}$. In addition, the serum concentrations of CRP during therapy were measured at 12 h, 24 h, 48 h, 72 h and 120 h after the commencement of the rIL2 infusions.

The metastatic tumours were assessed by ultrasound, CT radiography and MRI scanning (4 weeks after the start of treatment and subsequently at monthly intervals). If the disease was static or had responded (partial response was defined as a reduction of greater than 50% in tumour measurements carried out in two perpendicular planes; complete response was no tumour detected by imaging modalities), further therapy was given, as described above, to a maximum of six cycles.

The patients data was grouped into (i) responders (partial or complete) and (ii) non-responders (stasis or progression of disease), to treatment. The pre-treatment CRP levels were compared using Fischers exact test, and the time courses analysed using a Mann-Whitney U test to compare mean values at each time interval.

### Results

Six patients responded (1 complete and 5 partial responders) and 9 patients failed to demonstrate any response, to rIL2 therapy; their relevant details and serum concentrations of CRP, before treatment was started, are shown in Table I. The 95th percentile for serum CRP levels in normal individuals in our laboratory is $10\ mg\,l^{-1}$, and Table II shows that all responders had levels of less than $10\ mg\,l^{-1}$ range, $<2-8\ mg\,l^{-1}$, and that seven out of eight non-responders had serum concentrations of greater than $10\ mg\,l^{-1}$ (range, $8-116\ mg\,l^{-1}$), $P = 0.004$.

The time-course for the two groups, responders and non-responders, is shown in Figure 1. These are expressed as a Stimulation Index (SI) for time 't', (e.g. 12–72 h)

$$SI = \frac{\text{CRP serum concentration at 't'}}{\text{pre-treatment CRP concentration}}$$

The responders demonstrated a SI which rose substantially throughout the rIL2 infusion, from a pretreatment value of 1 to $31.3 \pm 9.3$ (mean ± s.e.m.) at the end of the infusion. In comparison, the pretreatment SI in the non-responding patients was 1, and demonstrated a minimal increase only during treatment, and was still only $1.6 \pm 0.3$ (mean ± s.e.m.) at the end of the rIL2 infusion. At all time intervals when CRP was measured during rIL2 infusion, the SI was significantly higher in the responding patients than in the non-responding patients ($P = 0.014$). One of the non-responding patients, (GR), had a low CRP level prior to the start of therapy, but the time course values for the CRP

Correspondence: S.D. Heys, Department of Surgery, University Medical Buildings, Foresterhill, Aberdeen, AB9 2ZD, UK.
Received 30 September 1991; and in revised form 13 July 1992.

Table I Clinical details and pre-treatment serum CRP levels in patients treated with rIL2

| *Patient* | *Age* | *Sex* | *Site of metastases* | *Response to rIL2* | *Pre-treatment CRP (mg l⁻¹)* |
|---|---|---|---|---|---|
| JW | 63 | m | liver | no response | 52 |
| JW | 54 | m | liver | complete response | 8 |
| DM | 70 | m | liver | no response | 116 |
| GR | 53 | m | liver | no response | 8 |
| JC | 63 | f | lymph nodes | partial response | 3 |
| FH | 52 | m | liver | no response | 51 |
| EQ | 73 | f | liver | no response | 115 |
| EB | 69 | f | liver | partial response | 7 |
| AJ | 65 | m | liver | no response | 63 |
| ET | 60 | f | lymph nodes | partial response | 4 |
| IW | 58 | m | liver | no response | 74 |
| JJ | 68 | m | liver | no response | 45 |
| GM | 38 | f | lymph nodes | partial response | 2 |
| GK | 68 | m | liver | partial response | 4 |
| CS | 38 | m | liver | no response | 110 |

Table II CRP concentrations in the serum of patients treated with rIL2

| | *CRP (>10 mg l⁻¹)* | *CRP (<10 mg l⁻¹)* |
|---|---|---|
| Responders ($n = 6$) | 0 | 6 |
| Range (mg l⁻¹) | – | <2–8 |
| Non-responders ($n = 9$) | 8 | 1 |
| Range (mg l⁻¹) | 45–116 | 8 |

**Figure 1** The time course for the CRP response during rIL2 infusion for 120 h. Values shown are means ± s.e.m., with all time points being significantly higher in the responding patients.

levels during the rIL2 infusion were similar to the other non-responders.

### Discussion

The acute phase protein, CRP, is one of thirty or more proteins produced by the liver in response to tissue damage and inflammation associated with infections, chronic disease states and malignant disease (Weinstein *et al.*, 1984; Kushner, 1982). Although different roles, such as inflammatory mediators, scavengers and enzyme inhibitors, have been ascribed to some of these proteins, the precise function of CRP remains to be elucidated. Nevertheless, it is a sensitive and relatively specific marker for monitoring inflammatory conditions, particularly those associated with significant tissue damage. In malignant disease, a high or rising level of serum CRP has been associated with a large tumour volume and dissemination, and poor prognosis (Weinstein *et al.*, 1984). The cytokines, IL1, IL6 and tumour necrosis factor (TNF), are believed to play a crucial role in the control of acute phase protein synthesis in the liver, by gene regulation (Dinarello, 1984; Baumann *et al.*, 1987; Marinkovic *et al.*, 1989).

Our data show that a response (partial or complete) to rIL2 occurred only in those patients whose serum CRP was less than 10 mg l⁻¹. In contrast, patients who failed to respond (stasis or progression of disease) to rIL2 therapy had CRP values which were grossly elevated, with the exception of one patient who had baseline levels. The reasons for the substantial pre-treatment difference between responding and non-responding patients remain unclear. All patients in this study had metastatic adenocarcinoma and three of the six responding patients had comparable tumour loads to those of the non-responding group of patients. However, the high baseline levels of CRP may be a reflection of either tumour burden or biological aggressiveness and hence enhanced tumour cell turnover (no patient had evidence of concurrent infection). The elevated levels of CRP induced by rIL2 in the responders may be a measure of cytokine release, in particular TNF, and subsequent beneficial anti-cancer response. Indeed, Blay *et al.* (1990) showed a correlation between sustained production of TNF and clinical response to rIL2. It is also important to note that the non-responding patient who had low baseline CRP levels (which can occur in patients with extensive tumour deposits) conformed to the pattern shown by all other non-responders, i.e. in failing to demonstrate a CRP response during the rIL2 infusion.

Thus, in assessing patients as to their suitability for continuous intravenous rIL2 therapy, with its associated morbidity and expense, it is apparent that patients who are already demonstrating a significant acute phase response, as demonstrated by increased circulating concentrations of CRP, are unlikely to respond to this treatment. Whilst patients with low baseline levels of CRP, in conjunction with the ability to mount a substantially enhanced CRP response during rIL2 infusion, should be selected for active and possibly prolonged treatment. Further careful studies are needed to confirm these findings and to elucidate the possible underlying mechanisms of anti-cancer activity.

We are grateful to Eurocetus for supplying the rIL2.

### References

BAUMANN, H., ONORATO, V., GAULDIE, J. & JAHREIS, G.P. (1987). Distinct sets of acute phase plasma proteins are stimulated by separate human hepatocyte-stimulating factors and monokines in rat hepatoma cells. *J. Biol. Chem.*, **262**, 9756–9768.

BLAY, Y-V., FAVROT, M.C., NEGRIER, S., COMBARET, V., CHOUAIB, S., MERCATELLO, A., KAEMMERLEN, P., FRANKS, C.R. & PHILLIP, T. (1990). Correlation between clinical response to interleukin 2 therapy and sustained production of tumour necrosis factor. *Cancer Res.*, **50**, 2371–2374.

DINARELLO, C.A. (1984). Interleukin-1. *Rev. Infect. Dis.*, **6**, 51–95.

KUSHNER, I. (1982). The phenomonen of the acute phase response. Ann. N.Y. Acad. Sci., **389**, 39–48.

MARINKOVIC, S., JAHREIS, G.P., WONG, G.C. & BAUMANN, H. (1989). IL-6 modulates the synthesis of a specific set of acute phase plasma proteins *in vivo*. *J. Immunol.*, **142**, 808–812.

ROSENBERG, S.A., LOTZE, M.T., YANG, J.C., AEBERSOLD, P.M., LINEHAN, W.M., SEIPP, C.A. & WHITE, D.E. (1989). Experience with the use of high-dose interleukin-2 in the treatment of 652 cancer patients. *Ann. Surg.*, **210**, 474–485.

STERNBERG, J.C. (1977). A rate nephelometer for measuring specific proteins by immunoprecipitin reactions. *Clin. Chem.* **23**, 1456–1464.

WEINSTEIN, P.S., SKINNER, M., SIPE, J.D., LOKICH, J.J., ZAMCHECK, N. & COHEN, A.S. (1984). Acute-phase proteins or tumour markers: The role of SAA, SAP, CRP and CEA as indicators of metastasis in a broad spectrum of neoplastic diseases. *Scand. J. Immunol.*, **19**, 193–198.

WEST, W.H., TAUER, K.W., YANNELLI, J.R., MARSHALL, G.D., ORR, D.W., THURMAN, G.B. & OLDHAM, R.K. (1987). Constant infusion recombinant interluekin-2 in adoptive immunotherapy of advanced cancer. *N. Engl. J. Med.*, **316**, 898–905.